# Structural features of the mitogenome of the leafhopper genus *Cladolidia* (Hemiptera: Cicadellidae: Coelidiinae) and phylogenetic implications in Cicadellidae

**DOI:** 10.1002/ece3.8001

**Published:** 2021-08-05

**Authors:** Xianyi Wang, Jiajia Wang, Renhuai Dai

**Affiliations:** ^1^ Guizhou Provincial Key Laboratory for Agricultural Pest Management of the Mountainous Region Institute of Entomology Guizhou University Guiyang China

**Keywords:** Cicadellidae, heterogeneity, leafhopper, mitochondrial phylogenomics, structure

## Abstract

The first two complete mitogenomes of the leafhopper genus *Cladolidia* (*C*. *biungulata* and *C. robusta*) were sequenced and annotated to further explore the phylogeny of *Cladolidia*. Both the newly sequenced mitogenomes have a typical circular structure, with lengths of 15,247 and 15,376 bp and A + T contents of 78.2% and 78%, respectively. We identified a highly conserved genome organization in the two *Cladolidia* spp. through comparative analysis that included the following assessments: genome content, gene order, nucleotide composition, codon usage, amino acid composition, and tRNA secondary structure. Moreover, we detected the base heterogeneity of Cicadellidae mitogenomic data and constructed phylogenetic trees using the nucleotide alignments of 12 subfamilies of 58 leafhopper species. We noted a weak heterogeneity in the base composition among the Cicadellidae mitogenomes. Phylogenetic analyses showed that the monophyly of each subfamily was generally well supported in the family Cicadellidae; the main topology was as follows: (Deltocephalinae + (Treehoppers + ((Megophthalminae + (Macropsinae + (Hylicinae + (Coelidiinae +Iassinae)) + (Idiocerinae + (Cicadellinae + (Typhlocybinae + (Mileewinae + (Evacanthinae +Ledrinae)))))))))). Within Coelidiinae, phylogenetic analyses revealed that *C*. *biungulata* and *C*. *robusta* belong to Coelidiinae and the monophyly of *Cladolidia* is well supported. In addition, on the basis of complete mitogenome phylogenetic analysis and the comparison of morphological characteristics, we further confirm the genus *Olidiana* as a paraphyletic group, suggesting that the genus may need taxonomic revisions.

## Graphical abstract

We gathered 63 mitochondrial genome sequences for leafhopper representing all 12 subfamilies, including 2 newly sequenced mitochondrial genomes from Coelidiinae. The phylogenetic relationship of each subfamily of Cicadellidae was further confirmed.

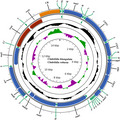

## 1 INTRODUCTION

In contrast to nuclear DNA, the mitogenome has a maternal mode of inheritance and is usually minimally recombinogenic; it carries genes with comparatively rapid evolutionary rates (Ballard & Whitlock, 2004; Cameron, 2014; Moritz & Brown, 1987; Wolstenholme, 1992). The entire mitogenome is a valuable source of extensive information compared with single genes. Moreover, it exhibits genome‐level characteristics, including gene content, base composition, gene organization, and gene secondary structure. These characteristics have been widely used for species identification as well as phylogenetic, phylogeographic, and genomic evolution studies (Anderson et al., 1981; Chuan et al., 2012; Nelson et al., 2012).

Leafhoppers are the members of a larger group of hemipterans and comprise >22,000 species (Dietrich, 2005). Recently, an increasing number phylogenic studies have been conducted on leafhoppers using mitogenomic data (Du, Dai, et al., 2017; Du, Zhang, et al., 2017; Du et al., 2019; Li et al., 2017; Song et al., 2017, 2019; Wang et al., 2020). So far, the data of 143 complete or near‐complete mitogenomes of Cicadellidae have been published in the National Center for Biotechnology Information (NCBI) database. Most of these organisms belong to the following subfamilies: Deltocephalinae (58), Cicadellinae (18), and Typhlocybinae (29). However, despite its vast diversity (>1,400 species), knowledge on the mitogenome of Coelidiinae is limited (Li & Fan, 2017; Nielson, 2015; Viraktamath & Meshram, 2019; Wang et al., 2018, 2021; Wang, Fan, et al., 2019; Zhang, 1990). Therefore, sequencing the mitogenomes of Coelidiinae may help enrich population genetics and phylogenetic studies regarding Cicadellidae (Hemiptera).

Most previous studies on Coelidiinae relationships have focused on morphological characteristics. However, the phylogeny of Coelidiinae remains to be explored using mitogenomic data. The lack of mitogenome sequences has limited the expansion of knowledge regarding the molecular evolution and population genetic diversity of this subfamily. Nielson (2015) removed *C*. *biungulata*, *C*. *robusta*, and five other species from *Calodia* and created the genus *Cladolidia* based primarily on the differences in the processes of aedeagus between these groupings. However, the position of the genus *Cladolidia* within the subfamily is yet to be ascertained (Nielson, 2015).

In the present study, we sequenced two complete mitogenomes of the genus *Cladolidia* (*C*. *biungulata* and *C*. *robusta*) using high‐throughput sequencing; *C*. *biungulata* and *C*. *robusta* are the first and second species, respectively, that have been described for this genus. In addition, we described their molecular phylogenetic relationships with 58 leafhopper and 5 treehopper species. Furthermore, this study provides an insight into the identification, phylogeny, conservation genetics, and evolution of *Cladolidia* and its related species.

## 2 MATERIALS AND METHODS

### 2.1 Sample collection and DNA extraction

Detailed information on the specimens collected is presented in Table S1. The collected specimens were identified based on their morphological characteristics, as described previously (Li & Fan, 2017; Zhang, 1990). After the species were accurately identified, the specimens were preserved in absolute ethanol and stored at −20°C until genomic DNA extraction. Genomic DNA was extracted from the whole body of adult males after removing the abdomen using DNeasy^®^ Blood & Tissue Kit. In brief, the samples were incubated at 56°C for 6 hr to lyse the cells completely and the total genomic DNA was eluted in 100‐μl double‐distilled water. The subsequent steps were performed according to the manufacturer's instructions. After evaluating the extracted genomic DNA quality using 1% agarose gel electrophoresis, it was stored at −20°C until further use. Both the voucher specimens with male genitalia and DNA samples have been deposited at the Institute of Entomology, Guizhou University, Guiyang, China.

### 2.2 Sequence analysis

The two complete mitogenomes of *C*. *biungulata* and *C*. *robusta* were sequenced by Berry Genomics on the HiSeq 2500 platform (Illumina) with 150‐bp paired‐end reads. The average insert length was 350 bp, and 6 GB of clean data were obtained. Each mitogenome was assembled using Geneious Prime 2019.2.1 software and based on a mitochondrial reference sequence of *Olidiana ritcheriina* (MK738125) (Wang, Wang, et al., 2019). The assembled mitochondrial gene sequences were compared with the homologous sequences of *O*. *ritcheriina* (MK738125) and *Taharana fasciana* (KY886913) (Wang et al., 2017; Wang, Wang, et al., 2019), which were retrieved from GenBank and identified via BLAST searches on NCBI to confirm sequence accuracy. We used the MITOS web server and BLAST searches on NCBI (https://blast.ncbi.nlm.nih.gov/Blast.cgi) to annotate the assembled sequences using invertebrate genetic codes (Altschul et al., 1997; Bernt et al., 2013) as well as the search server tRNAscan‐SE 1.21 to identify the locations and predict the secondary structure of 22 typical tRNAs (Laslett & Canbäck, 2008; Schattner et al., 2005; Tamura et al., 2013). All rRNA genes were identified based on the locations of adjacent tRNA genes and comparisons with sequences of other leafhopper mitogenomes deposited in NCBI. ORF Finder in Geneious Prime was used to predict 13 protein‐coding gene (PCG) locations using invertebrate genetic codes. The mitogenomic map and comparative analysis were performed using CGView comparison tool (Stothard et al., 2017). Furthermore, the relative synonymous codon usage (RSCU) values and codon numbers were calculated using the MEGA version 7.0 program (Sudhir et al., 2016). Finally, chain asymmetry was calculated using the following formulas: AT skew = (A − T)/(A + T) and GC skew = (G − C)/(G + C) (Perna & Kocher, 1995).

### 2.3 Phylogenetic analysis

In total, 58 leafhopper and 5 treehopper species were selected to construct the phylogenetic tree after the removal of sequences that were unverified, lacked an accurate scientific name, and were repetitive. Phylogenetic analysis was performed using alignments of the 13 PCGs of leafhopper species with the other complete or near‐complete mitogenomes of the treehopper species. The two species of *Cosmoscarta bispecularis* (KP064511) and *Tettigades auropilosa* (KM000129) (Yan & Zu, 2019) were used as the outgroup (Table S2). Each PCG was aligned using the TranslatorX online tool, employing MAFFT to perform protein alignment (Abascal et al., 2010; Castresana, 2000; Katoh et al., 2017). Then, the resulting 13 alignments were assessed and manually corrected using the MEGA version 7.0 program (Sudhir et al., 2016). The best schemes for partition and substitution models (Table S3) were determined in PartitionFinder version 2.1.1 using the Akaike information criterion and the greedy search algorithm (Lanfear et al., 2017). For phylogenetic analyses, the maximum likelihood (ML) and the Bayesian inference (BI) methods were used to construct the ML and BI trees based on two datasets (13PCG12, first and second codons of 13 PCGs [6,676 bp]; the amino acid [AA] sequences of 13 PCGs [3,338 bp]; these datasets were deposited in Dryad: https://doi.org/10.5061/dryad.zkh1893b3). The third codon positions may suffer from mutation saturation, which can lead to noise in the phylogenetic analysis (Blouin et al., 1998; Breinholt & Kawahara, 2013). Hence, the third codons were discarded from the phylogenetic analysis. The heterogeneity of sequence divergence within the two datasets was analyzed using AliGROOVE, with the default sliding window size (Kück et al., 2014).

ML analysis was performed with 1,000 rapid bootstrapping replicates using iqtree (Suchard & Huelsenbeck, 2012), whereas BI analysis was performed in MrBayes 3.2.7a with 4 chains and sampling of the chains every 1,000 generations (Nguyen et al., 2014). Two independent runs of 10 million generations were performed. After the average standard deviation of split frequencies fell to <0.001, the initial 25% of the samples was discarded as burn‐in and the remaining trees were used to generate a consensus tree and calculate the posterior probabilities. The BI and ML analyses were performed on the CIPRES Science Gateway (https://www.phylo.org) website. The phylogenetic trees were visualized using FigTree 1.4.2.

## 3 RESULTS

### 3.1 General features of *Cladolidia* mitogenome

**FIGURE 1:**
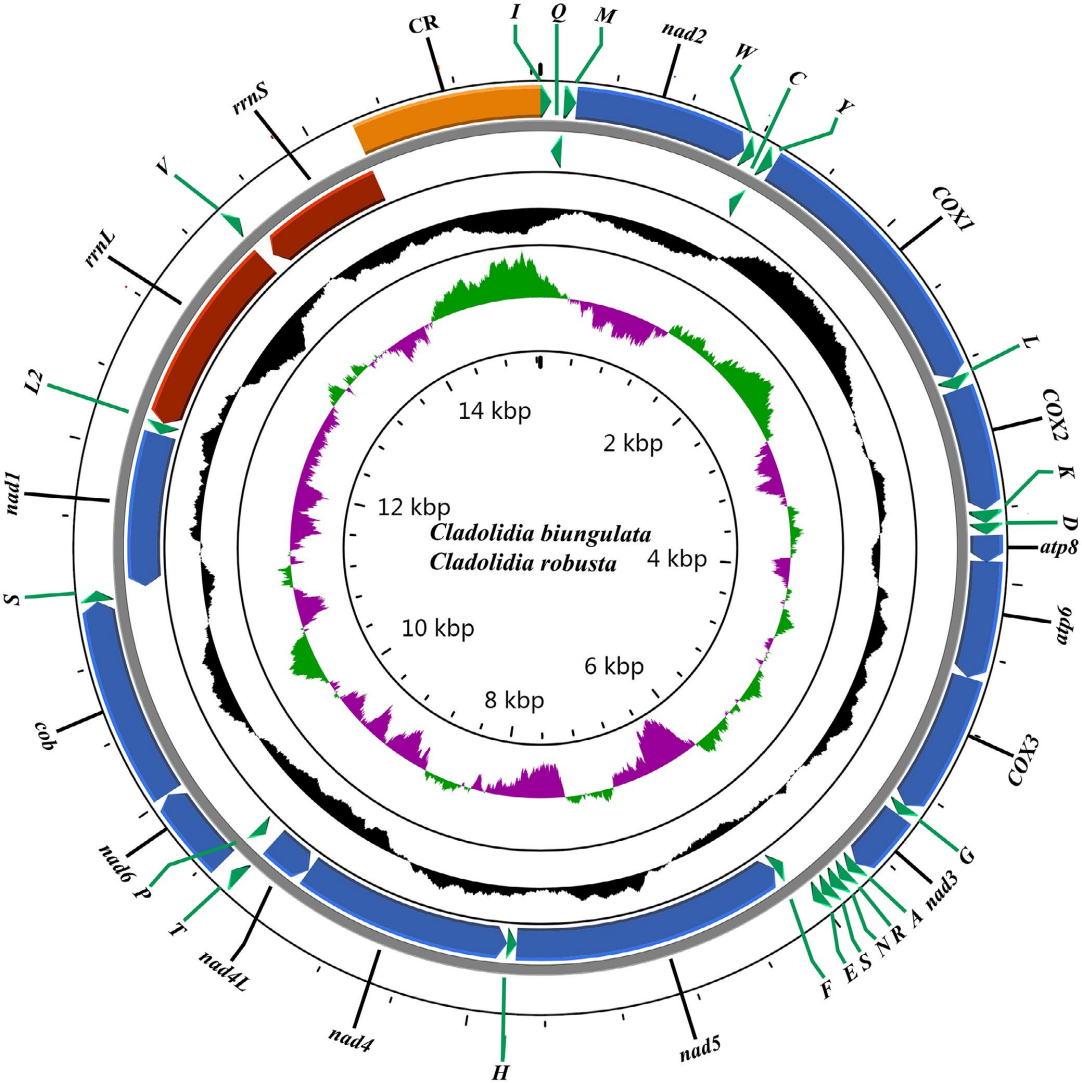
Mitogenome map of *Cladolidia* spp

The annotations of the mitogenomes of the two *Cladolidia* species and the circular maps are shown in Table 1 and Figure 1, respectively. The two complete mitogenome sequences of *C*. *biungulata* (MW406474) and *C*. *robusta* (MW406475) are closed‐circular molecules, with lengths of 15,247 and 15,376 bp, respectively. These completely sequenced mitogenomes are medium‐sized in length and within the range of those of other Cicadellidae species (14,805 bp of *Nephotettix cincticeps* to 17,562 bp of *Parazyginella tiani*) (Wang et al., 2018). The two mitogenomes contained a typical set of 37 mitochondrial genes (13 PCGs, 22 tRNAs, and 2 rRNAs) along with a control region. Of these 37 genes, 23 are present on the heavy strand (J‐strand), whereas 14 are located on the light strand (N‐strand) (Figure 1, Table 1). The gene order of these two mitogenomes is identical to that of all previously published mitogenomes of Cicadellidae and the ancestral *Drosophila yakuba* (Clary & Wolstenholme, 1985). These two mitogenomes of *Cladolidia* contain 10 nucleotides that are dispersed among six intergenic spacers (ranging from 1 to 4 bp), and the longest spacer sequence (4 bp) is located between *trnH* and *nad5*, *trnA*, and *trnR*. There are a total of 14 overlapping regions (ranging from 1 to 11 bp), and the conserved 11‐bp overlapping nucleotide sequence between *trnW* and *trnC* is extremely common in Cicadellidae (Du, Zhang, et al., 2017; Du et al., 2019; Wang et al., 2017, 2018, 2020, 2021; Wang, Wang, et al., 2019).

**TABLE 1:** Organization of the *Cladolidia robusta*/*C*. *biungulata* mitogenome

Gene	Direction	Length (bp)	Start	Stop	Anticodon	Intergenic nucleotides	AT content (%)
*trnI*	J	62	–	–	GAT		77.4
*trnQ*	N	67	–	–	TTG	1	79.1/77.6
*trnM*	J	68/66	–	–	CAT	−1/0	75/74.2
*nad2*	J	955/957	ATT	T/TAA	–	0	82.1/82.7
*trnW*	J	62	–	–	TCA	0/−2	80.680.6
*trnC*	N	57/65	–	–	GCA	−8/−11	84.2/84.6
*trnY*	N	63/62	–	–	GTA	0/−5	79.4/79
*cox1*	J	1,536	ATG	TAA	–	2	71.5/72.5
*trnL1(UUR)*	J	67/68	–	–	TAA	0	82.1/82.4
*cox2*	J	676	ATT	T	–	0	76.5/75.9
*trnK*	J	71	–	–	CTT	0	76.1/77.5
*trnD*	J	64	–	–	GTC	−1/0	85.9/84.4
*atp8*	J	150	ATA	TAA	–	1/0	82/82.7
*atp6*	J	636	ATA	TAA	–	−1	76.3/77.7
*cox3*	J	778	ATG	T	–	0	73.5/73.8
*trnG*	J	61/63	–	–	TCC	0/−2	75.4/79.4
*nad3*	J	354	ATA	TAG	–	0	79.1/80.4
*trnA*	J	61	–	–	TGC	−2	80.3
*trnR*	J	59/63	–	–	TCG	4/1	74.6
*trnN*	J	64	–	–	GTT	−1/−2	78.1/76.6
*trnS1*	J	62	–	–	GCT	−1	7,169.4
*trnE*	J	63	–	–	TTC	−1	87.3/87.3
*trnF*	N	67	–	–	GAA	−1	82.1/83.6
*nad5*	N	1,674	ATT	TAA	–	−1	77.4/77.9
*trnH*	N	60	–	–	GTG	0	75/78.3
*nad4*	N	1,308	ATT/ ATG	TAA	–	−1	77.8/78
*nad4l*	N	276	ATG	TAA/TAG	–	2	83.7/84.4
*trnT*	J	65	–	–	TGT	2	87.7
*trnP*	N	62	–	–	TGG	0	74.2/75.8
*nad6*	J	474	ATT	TAA	–	4/2	82.3/80.8
*cob*	J	1,122/1,126	ATA/ATC	TAA	–	0	73.5/74.1
*trnS2(UCN)*	J	61/64	–	–	TGA	−1/−1	82/79.7
*nad1*	N	939	ATT	TAA	–	−4/−7	77.2/78.4
*trnL2(CUN)*	N	68	–	–	TAG	0	75/79.4
*rrnL*	N	1,186/1,182	–	–	–	0	82/82.2
*trnV*	N	60	–	–	TAC	0	73.3/75
*rrnS*	N	779/730	–	–		0	81.6/81.5
CR		1,016/1,199	–	–	–	0	84/82.5

The nucleotide composition of the two *Cladolidia* species reveals a strong A + T bias in the entire mitogenome, and the A + T contents between *C*. *biungulata* and *C*. *robusta* are nearly equal (78.2% in *C*. *biungulata* and 78.4% in *C*. *robusta*). As with other Coelidiinae, the nucleotide composition of the two mitogenomes is clearly biased toward A/T nucleotides, with 13 PCGs, 22 tRNAs, <2 rRNAs, and a control region. This phenomenon to some extent is due to the damage or accumulation of mutations in the mitochondrial DNA (Martin, 1995).

### 3.2 PCGs and codon usage of *Cladolidia* mitogenome

A total of 13 PCGs were identified in each of the two *Cladolidia* mitogenomes. In both mitogenomes, all PCGs use the canonical initiation codon ATN and the canonical stop codon TAA/TAG, except for *cox2* and *cox3*. *C*. *biungulata* also harbors *nad2*, which uses an incomplete stop codon T‐‐. This phenomenon has also been noted in other Coelidiinae insects (Wang et al., 2017; Wang, Wang, et al., 2019). The incomplete stop codons are modified into complete TAA codons via posttranscriptional polyadenylation during mRNA maturation (Perna & Kocher, 1995). Of note, *cox1*, *cox3*, and *atp6* in each species have the same start and stop codons. The longest PCG is *nad5* (1,674 bp), and the shortest is *atp8* (150 bp). Only four genes (*nad5*, *nad4*, *nad4l*, and *nad1*) are present on the N‐strand. The other nine genes (*cox1*, *cox2*, *cox3*, *atp8*, *atp6*, *nad2*, *nad3*, *nad6*, and *cob*) are located on the J‐strand (Figure 1, Table 1), which is similar to the mitogenome structure of most other Coelidiinae insects (Wang et al., 2017, 2021; Wang, Wang, et al., 2019).

**FIGURE 2:**
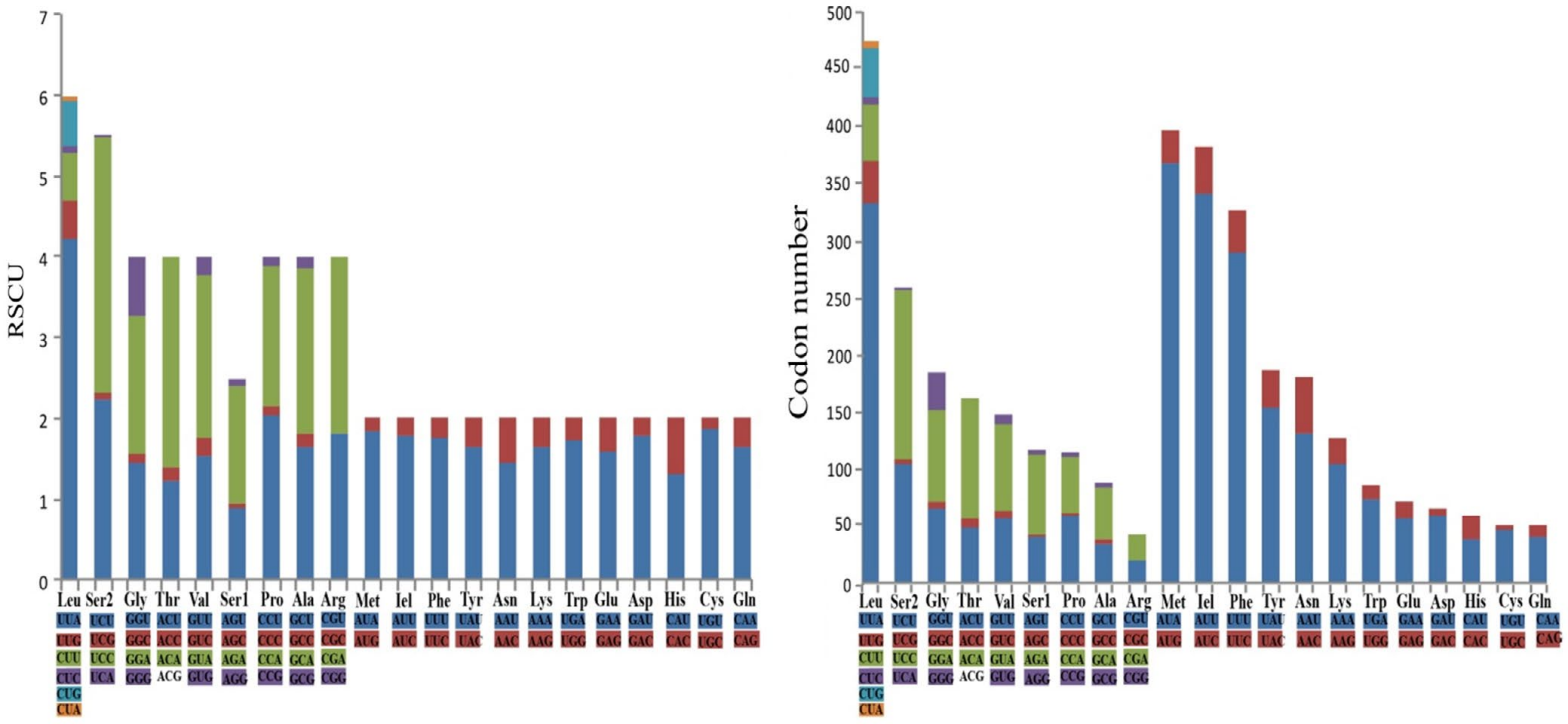
Relative synonymous codon usage (RSCU) and codon number of *Cladolidia robusta*

The RSCU values and codon number for *C*. *robusta* (very similar to *C*. *biungulata*) are shown in Figure 2. The most frequently used codon is AUA (Met, *N* = 367), followed by AUU (Ile, *N* = 340), UUA (Leu, *N* = 333), and UUU (Phe, *N* = 290). However, in previous studies, the most frequently used codon was UUU (Phe) (Wang et al., 2017, 2021; Wang, Wang, et al., 2019). Moreover, the majority of frequently used codons end with A or U (Figure 2). These two factors appear to contribute to the high A + T content of PCGs and the AT bias of the whole mitogenome.

**FIGURE 3:**
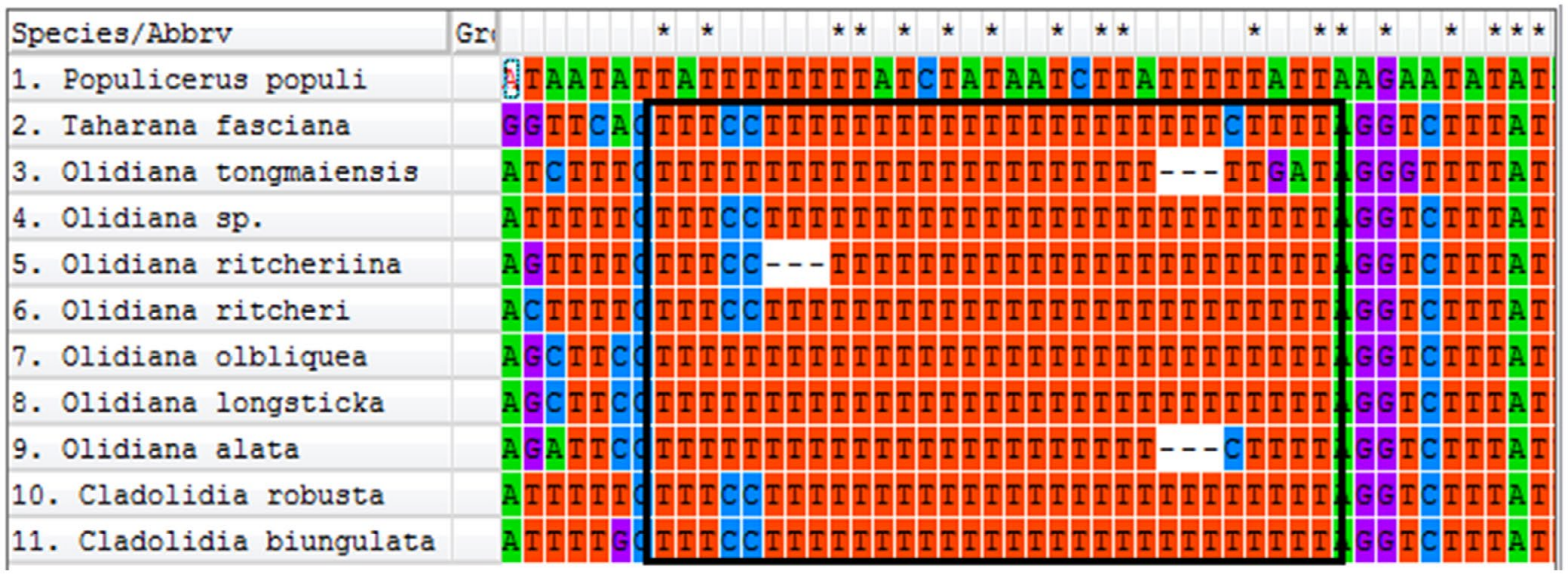
Poly‐T structure of ND5 in the subfamily Coelidiinae

Comparative analysis revealed that the mitogenome of Coelidiinae is a conservative poly‐T (with 28–31 bp) structure (Figure 3). Such a large poly‐T structure is not found in the mitogenomes of other leafhoppers; hence, we hypothesized that this particular structure serves as a DNA barcode for the subfamily.

### 3.3 tRNAs and rRNAs of *Cladolidia* mitogenome

**FIGURE 4:**
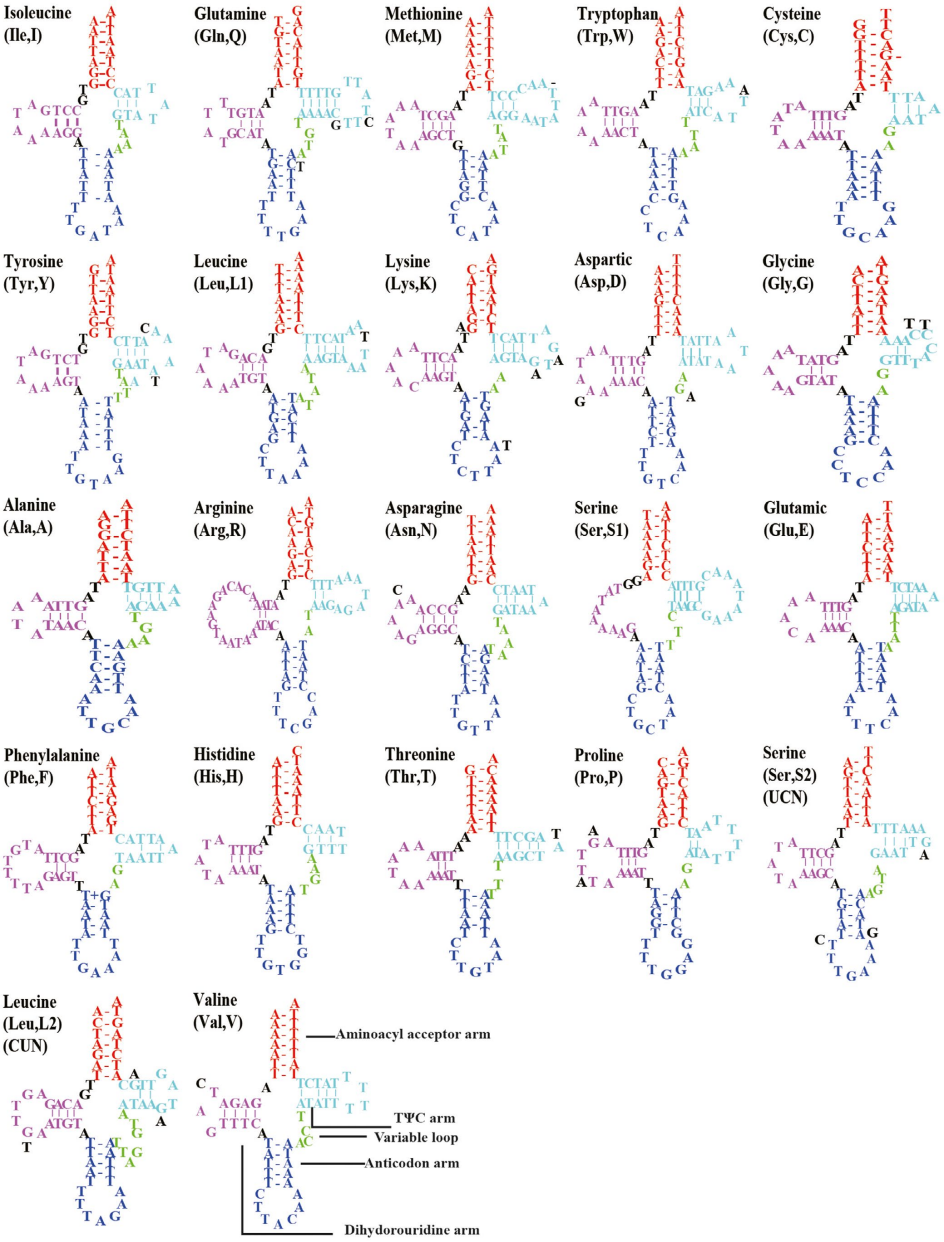
Predicted secondary structures of the 22 tRNAs of *Cladolidia biungulata* mitogenome. “‐” indicates the sites without a codon in *Cladolidia robusta*

All 22 tRNAs of *C*. *biungulata* and *C*. *robusta* mitogenomes were identified; they ranged from 57 to 68 bp in length. Among the tRNA genes, 14 are located on the J‐strand and 8 on the N‐strand, which is the coding pattern observed in almost all Cicadellidae mitogenomes (Du, Zhang, et al., 2017; Du et al., 2019; Wang et al., 2017, 2018, 2020, 2021; Wang, Wang, et al., 2019). The 22 tRNA genes in the two *Cladolidia* species were identified, and their secondary structures are shown in Figure 4. All these gene products are folded into the typical cloverleaf secondary structure, except *trnS1*, which lacks the dihydrouridine (DHU) arm; the loss of the DHU arm in *trnS1* is a typical feature in Cicadellidae mitogenomes (Wang et al., 2017, 2018; Wang, Wang, et al., 2019). The combined length of tRNA genes of *C*. *biungulata* and *C*. *robusta* is 1,411 bp and 1,394 bp, with A + T contents of 79.4% and 78.9%, respectively. *rrnS* is located between *trnL2* (CUN) and *trnV*, whereas *rrnL* is flanked by *trnV* and the control region (Figure 1, Table 2). Two rRNA genes, *rrnS* and *rrnL*, in *C*. *biungulata* and *C*. *robusta* have the same total length (2,222 bp). In *Cladolidia*, the A + T (81.8%) contents are the same and AT skews can be either positive or negative. The 22 tRNA and 2 rRNA genes are highly conserved, particularly *trnI*, *trnA*, *trnR*, *and trnE*, and the secondary structures are exactly the same between *C*. *biungulata* and *C*. *robusta*.

**TABLE 2:** Nucleotide composition and skewness of *Cladolidia* mitogenomes

	Region	Length	AT content%	AT skew	GC skew
*Cladolidia biungulata*	Whole	15,376	78.2	0.16	−0.24
	13 PCGs	1,411	79.4	0.18	−0.26
	22 tRNAs	1,394	78.9	0.12	−0.14
	2 rRNAs	1,972	81.8	0.18	−0.27
	Control region	1,199	83	−0.02	−0.01
*Cladolidia robusta*	Whole	15,247	78.4	0.16	−0.24
	13 PCGs	1,394	78.9	0.18	−0.25
	22 tRNAs	1,402	79.5	0.11	−0.10
	2 rRNAs	1,956	81.8	0.17	−0.27
	Control region	1,016	82.5	0.01	−0.09

Abbreviation: PCG, protein‐coding gene.

### 3.4 Control region of *Cladolidia* mitogenome

The control regions are located between *rrnS* and *trnI*, with lengths of 1,016 (*C*. *biungulata*) and 1,199 bp (*C*. *robusta*), respectively. The control region has the highest A + T content (83% and 82.5%) among the two complete *C*. *biungulata* and *C*. *robusta* mitogenomes (Table 2). Comparative analysis of the base composition of every component of the Coelidiinae mitogenomes indicated that the control regions have the highest A + T content, ranging from 82.5% (*C*. *robusta*) to 85.9% (*O*. *obliqua*). In the control region, both AT and GC skew are negative, indicating that T and C are more abundant than A and G. The GC content was the most significant factor in determining the codon bias among organisms, which is consistent with the general tendency of the complete mitogenome.

### 3.5 Phylogenetic relationship

**FIGURE 5:**
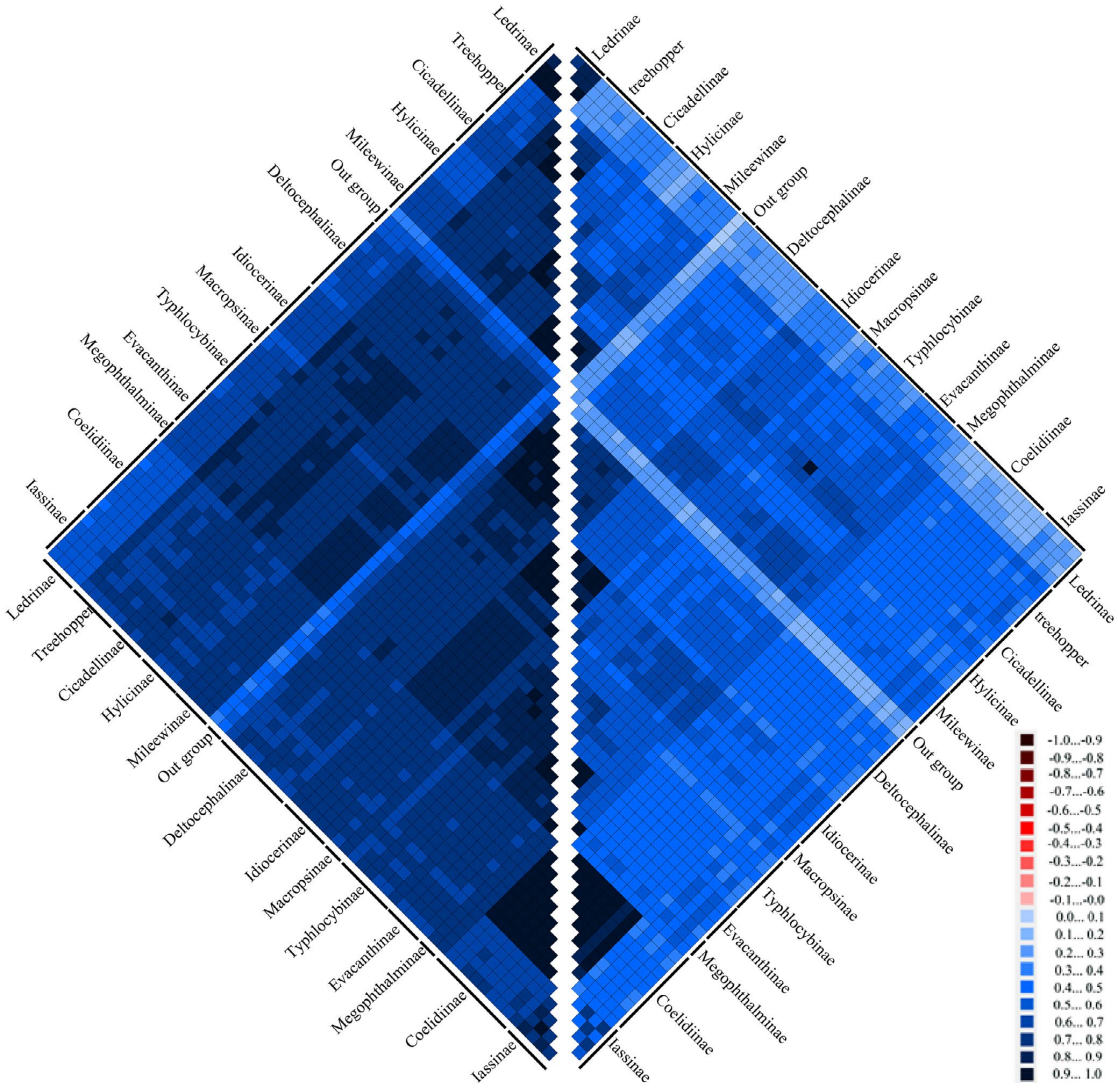
Heterogeneity of amino acids (left) and PCG12 (right) in the mitogenome of Cicadellidae. Differences in heterogeneity between sequences are represented by color, with dark red (−1) to dark blue (+1) representing differences from heavy to light. PCG, protein‐coding gene

By detecting the base heterogeneity of mitogenome datasets used for constructing a phylogenetic tree, we can determine whether the base heterogeneity of each dataset will cause a major error in the tree construction process (Li et al., 2015; Liua et al., 2018; Morgan et al., 2013; Sheffield et al., 2009; Song et al., 2016; Timmermans et al., 2015). On the basis of the calculation results obtained from the AliGROOVE (Kück et al., 2014) software, the heterogeneity of PCG12 and AA datasets in the mitogenomic data of Cicadellidae is weak (Figure 5). Hence, the two datasets could be used to construct a phylogenetic tree.

**FIGURE 6:**
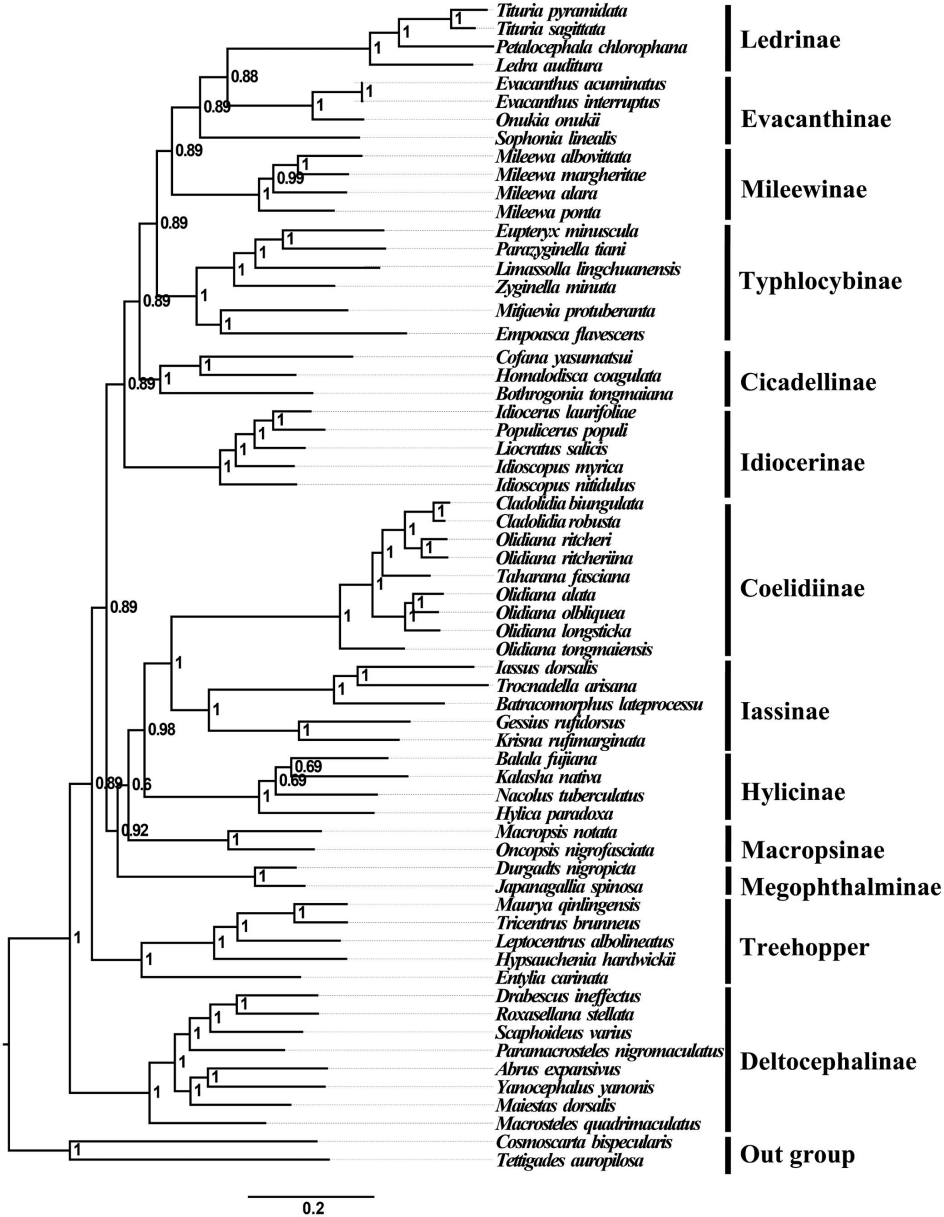
Phylogenetic tree of Cicadellidae species inferred via Bayesian analyses of the amino acid datasets

**FIGURE 7:**
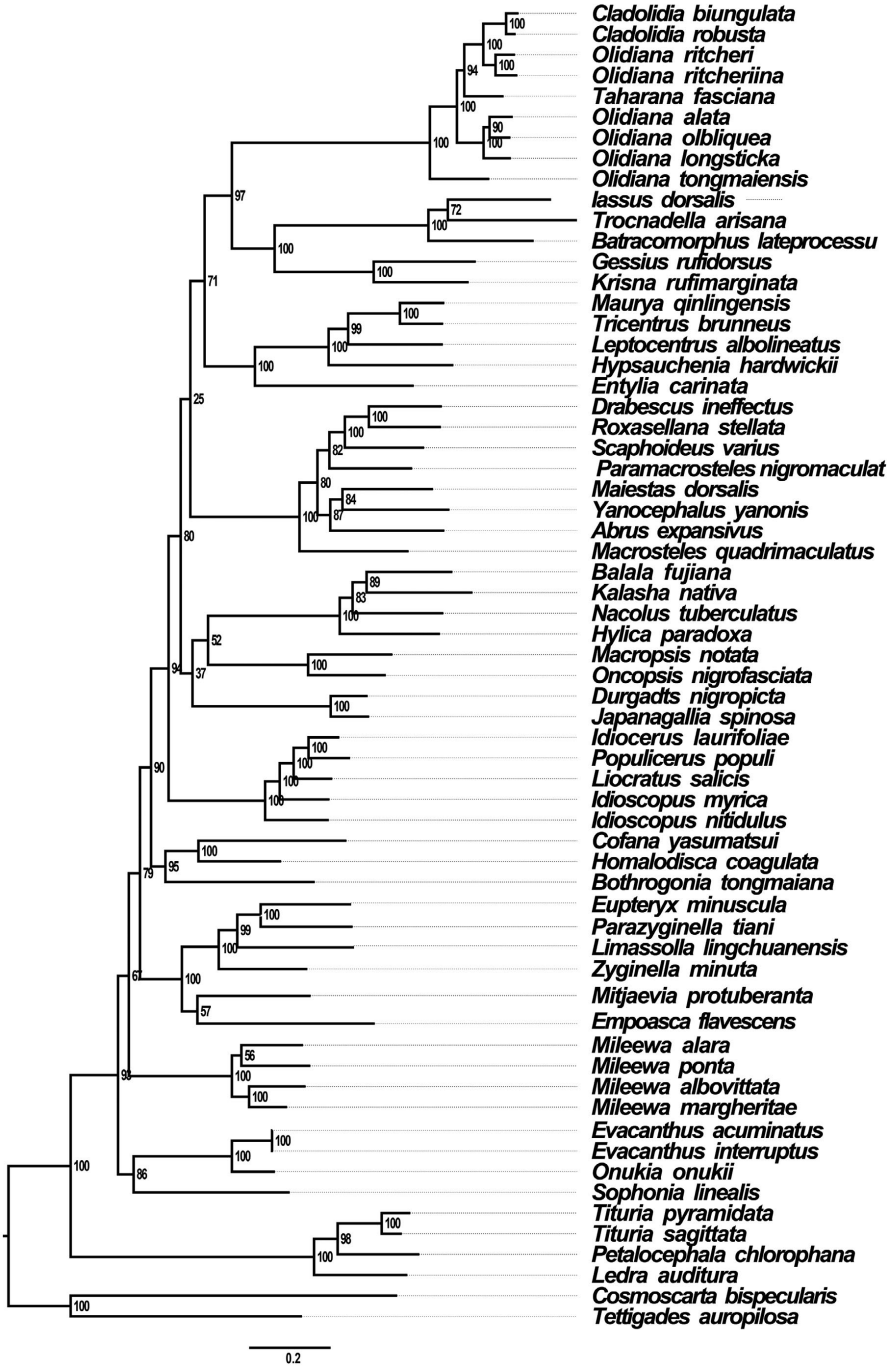
Phylogenetic tree Cicadellidae species inferred via maximum likelihood analyses of the amino acid datasets

BI and ML analyses using 13PCGs12 and the AA datasets generated phylogenic trees with two topologies (Figures 6, 7, S8, and S9). The monophyly of each subfamily was generally well supported in the family Cicadellidae, which is consistent with the findings of some previous molecular phylogenetic studies (Du, Zhang, et al., 2017; Du et al., 2019; Wang et al., 2017, 2018, 2020, 2021; Wang, Wang, et al., 2019). However, this finding is different from that of the studies by (Xue et al., 2020) and (Dietrich et al., 2017). They supported the inclusion of Macropsini and Idiocerini as the tribes of Eurymelinae. The phylogenetic relationships determined in our study do not support this inclusion, possibly owing to the use of molecular data different from those of previous studies and the limited mitogenomic data evaluation in our study; therefore, multiple gene types and more taxa should be sampled in the future to resolve this issue. Our analyses confirm that Iassinae is a sister group of Coelidiinae. Nine species of Coelidiinae are clustered together, and all phylogenetic relationships demonstrated a high nodal support in both ML (bootstrap support [BS] > 90) and BI (posterior probabilities [PP] = 1.00) analyses. These results provide substantial support for these two species (*C*. *biungulata* and *C*. *robusta*) being the members of the Coelidiinae subfamily and Cicadellidae family.

## 4 DISCUSSION

The results of all analyses performed in the present study clearly support the 12 included cicadellidae subfamilies being monophyletic groups. The BI tree showed the following relationship within Membracoidea: (Deltocephalinae + (Treehoppers + ((Megophthalminae + (Macropsinae + (Hylicinae + (Coelidiinae +Iassinae)) + (Idiocerinae + (Cicadellinae + (Typhlocybinae + (Mileewinae + (Evacanthinae +Ledrinae))))))))) (Figure 6, S8, and S9). However, the ML tree showed the following phylogenetic relationships: (Ledrinae + (Evacanthinae + (Mileewinae + (Typhlocybinae + (Cicadellinae + (Idiocerinae + ((Macropsinae + (Megophthalminae +Hylicinae)+ (Deltocephalinae + ((Treehoppers + (Coelidiinae +Iassinae) +))))))))))) (Figure 7). In all BI analyses with higher approval ratings than ML analyses, this phenomenon is commonly noted in the analyses performed in previous studies; other recent analyses of relationships among some leafhopper subfamilies have yielded trees with low support for many deep internal branches (Wang et al., 2018, 2020). These two relationships of BI analyses and ML analyses differ primarily in the positions of Deltocephalinae and Ledrinae. In ML‐AA analysis, Ledrinae occupied the basal branch of leafhopper species in all phylogenetic analyses. This further confirms that the subfamily Ledrinae is an ancient group of leafhoppers, which is consistent with the findings of previous molecular phylogenetic studies (Du, Zhang, et al., 2017; Du et al., 2019; Wang et al., 2017, 2018, 2020, 2021; Wang, Wang, et al., 2019). However, Deltocephalinae, rather than Ledrinae, occupied the basal branch of leafhopper species in other (BI‐AA, BI/ML‐PCG12) phylogenetic analyses. Our analyses confirm that Iassinae and Coelidiinae are assigned to the sister groups of treehoppers, Macropsinae, and Megophthalminae with high approval ratings (ML, BS = 100; BI, PP = 1.00); this result is different from that observed in previous studies (Du et al., 2019; Wang et al., 2017, 2018, 2020, 2021; Wang, Wang, et al., 2019). In the present study, phylogenetic relationships showed that the subfamily Megophthalminae is a sister group of Macropsinae instead of treehoppers.

In all analyses, the two species of the genus *Cladolidia* also clustered closely with the genus *Taharana*; the results showed that the genus *Cladolidia* is a monophylic group. However, the genus *Olidiana* was not classified as monophyletic and can be divided into three branches. The three species *O*. *ritcheriina*, *Olidiana* sp., and *O*. *ritcheri* also clustered closely to the genus *Taharana*. The remaining species were split into two clades: one included *O*. *longsticka*, *O*. *obliqua*, and *O*. *alata* and the other included only one species (*O*. *tongmaiensis*). This conclusion was further confirmed based on significant differences in their morphological characteristics, which were characterized by body color, shape, and the position of the processes on the aedeagus shaft. Therefore, on the basis of the complete mitogenome phylogenetic analysis and the comparison of morphological characteristics, we propose that *Olidiana* is not monophyletic; hence, this genus may need taxonomic revisions. Future studies on both the morphological and molecular characteristics of additional species are warranted to reveal phylogenetic relationships within Coelidiinae.

## CONFLICTS OF INTEREST

The authors declare no conflicts of interest.

## AUTHOR CONTRIBUTIONS

**Xianyi Wang:** Formal analysis (equal); Investigation (lead); Methodology (lead); Writing‐original draft (equal); Writing‐review & editing (equal). **Jiajia Wang:** Conceptualization (equal); Investigation (equal); Methodology (equal); Writing‐review & editing (equal). **Renhuai Dai:** Conceptualization (equal); Formal analysis (equal); Writing‐original draft (equal); Writing‐review & editing (equal).

## Supporting information

Appendix S1

## Data Availability

GenBank accession numbers: *Cladolidia biungulata* (MW406474) and *Cladolidia robusta* (MW406475). These two datasets 13PCG12 dataset (first and second codons of 13 PCGs, 6,676 bp); AA dataset, the amino acid sequences of 13 PCGs, 3,338 bp were deposited in Dryad: https://doi.org/10.5061/dryad.zkh1893b3).

## References

[ece38001-bib-0001] Abascal, F., Zardoya, R., & Telford, M. J. (2010). TranslatorX: Multiple alignment of nucleotide sequences guided by amino acid translations. Nucleic Acids Research, 38(Web Server), W7–W13. 10.1093/nar/gkq291 20435676 PMC2896173

[ece38001-bib-0002] Altschul, S. F., Madden, T. L., Schaffer, A. A., Zhang, J., Zhang, Z., Miller, W., & Lipman, D. J. (1997). Gapped BLAST and PSI‐BLAST: A new generation of protein database search programs. Nucleic Acids Research, 25, 3389–3402. 10.1093/nar/25.17.3389 9254694 PMC146917

[ece38001-bib-0003] Anderson, S., Bankier, A. T., Barrell, B. G., de Bruijn, M. H. L., Coulson, A. R., Drouin, J., Eperon, I. C., Nierlich, D. P., Roe, B. A., Sanger, F., Schreier, P. H., Smith, A. J. H., Staden, R., & Young, I. G. (1981). Sequence and organization of the human mitochondrial genome. Nature, 290, 457–465. 10.1038/290457a0 7219534

[ece38001-bib-0004] Ballard, J. W., & Whitlock, M. C. (2004). The incomplete natural history of mitochondria. Molecular Ecology, 13, 729–744. 10.1046/j.1365-294X.2003.02063.x 15012752

[ece38001-bib-0005] Bernt, M., Donath, A., Jühling, F., Externbrink, F., Florentz, C., Fritzsch, G., Pütz, J., Middendorf, M., & Stadler, P. F. (2013). MITOS: Improved de novo metazoan mitochondrial genome annotation. Molecular Phylogenetics and Evolution, 69, 313–319. 10.1016/j.ympev.2012.08.023 22982435

[ece38001-bib-0006] Blouin, M. S., Yowell, C. A., Courtney, C. H., & Dame, J. B. (1998). Substitution bias, rapid saturation, and the use of mtDNA for nematode systematics. Molecular Biology and Evolution, 15, 1719–1727. 10.1093/oxfordjournals.molbev.a025898 9866206

[ece38001-bib-0007] Breinholt, J. W., & Kawahara, A. Y. (2013). Phylotranscriptomics: Saturated third codon positions radically influence the estimation of trees based on next‐gen data. Genome Biology and Evolution, 5, 2082–2092. 10.1093/gbe/evt157 24148944 PMC3845638

[ece38001-bib-0008] Cameron, S. L. (2014). Insect mitochondrial genomics: implications for evolution and phylogeny. Annual Review of Entomology, 59, 95–117. 10.1146/annurev-ento-011613-162007 24160435

[ece38001-bib-0009] Castresana, J. (2000). Selection of conserved blocks from multiple alignments for their use in phylogenetic analysis. Molecular Biology and Evolution, 17, 540–552. 10.1093/oxfordjournals.molbev.a026334 10742046

[ece38001-bib-0010] Chuan, M. A., Yang, P., Jiang, F., Chapuis, M. P., Shali, Y., Sword, G. A., & Kang, L. E. (2012). Mitochondrial genomes reveal the global phylogeography and dispersal routes of the migratory locust. Molecular Ecology, 21, 4344–4358. 10.1111/j.1365-294X.2012.05684.x 22738353

[ece38001-bib-0011] Clary, D. O., & Wolstenholme, D. R. (1985). The mitochondrial DNA molecule of *Drosophila yakuba*: Nucleotide sequence, gene organization, and genetic code. Journal of Molecular Evolution, 22, 252–271. 10.1007/BF02099755 3001325

[ece38001-bib-0012] Dietrich, C. H. (2005). Keys to the families of Cicadomorpha and subfamilies and tribes of Cicadellidae (Hemiptera: Auchenorrhyncha). Florida Entomologist, 88, 502–517. 10.1007/0-387-31311-7_34

[ece38001-bib-0051] Dietrich, C. H., Allen, J. M., Lemmon, A. R., Lemmon, E. M., Takiya, D. M., Evangelista, O. W., & Johnson, K. P. (2017). Anchored hybrid enrichment‐based phylogenomics of leafhoppers and treehoppers (Hemiptera: Cicadomorpha: Membracoidea). Insect Systematics and Diversity, 1, 57–72. 10.1093/isd/ixx003

[ece38001-bib-0013] Du, Y., Dai, W., & Dietrich, C. H. (2017). Mitochondrial genomic variation and phylogenetic relationships of three groups in the genus *Scaphoideus* (Hemiptera: Cicadellidae: Deltocephalinae). Scientific Reports, 7, 14197. 10.1038/s41598-017-17145-z 29203807 PMC5714952

[ece38001-bib-0014] Du, Y. M., Dietrich, C. H., & Dai, W. (2019). Complete mitochondrial genome of *Macrosteles quadrimaculatus* (Matsumura) (Hemiptera: Cicadellidae: Deltocephalinae) with a shared tRNA rearrangement and its phylogenetic implications. International Journal of Biological Macromolecules, 122, 1027–1034. 10.1016/j.ijbiomac.2018.09.049 30218730

[ece38001-bib-0015] Du, Y., Zhang, C., Dietrich, C. H., Zhang, Y., & Dai, W. (2017). Characterization of the complete mitochondrial genomes of *Maiestas dorsalis* and *Japananus hyalinus* (Hemiptera: Cicadellidae) and comparison with other Membracoidea. Scientific Reports, 7, 14197. 10.1038/s41598-017-14703-3 29079765 PMC5660246

[ece38001-bib-0016] Katoh, K., Rozewicki, J., & Yamada, K. D. (2017). MAFFT online service: Multiple sequence alignment, interactive sequence choice and visualization. Briefings in Bioinformatics, 4, 1–7. 10.1093/bib/bbx108 PMC6781576 28968734

[ece38001-bib-0017] Kück, P., Meid, S. A., Groß, C., Wägele, J. W., & Misof, B. (2014). AliGROOVE – visualization of heterogeneous sequence divergence within multiple sequence alignments and detection of inflated branch support. BMC Bioinformatics, 15, 294. 10.1186/1471-2105-15-294 25176556 PMC4167143

[ece38001-bib-0018] Lanfear, R., Frandsen, P. B., Wright, A. M., Senfeld, T., & Calcott, B. (2017). Partitionfinder 2: New methods for selecting partitioned models of evolution for molecular and morphological phylogenetic analyses. Molecular Biology and Evolution, 34, 772–773. 10.1093/molbev/msw260 28013191

[ece38001-bib-0019] Laslett, D., & Canbäck, B. (2008). ARWEN: A program to detect tRNA genes in metazoan mitochondrial nucleotide sequences. Bioinformatics, 24, 172–175. 10.1093/bioinformatics/btm573 18033792

[ece38001-bib-0020] Li, H., Leavengood, J. M., Chapman, E. G., Burkhardt, D., Song, F., Jiang, P., Liu, J. P., Zhou, X. G., & Cai, W. Z. (2017). Mitochondrial phylogenomics of Hemiptera reveals adaptive innovations driving the diversification of true bugs. Proceedings of the Royal Society. Series B. Biological Sciences, 284, 20171223. 10.1098/rspb.2017.1223 PMC5597834 28878063

[ece38001-bib-0021] Li, H., Shao, R. F., Song, N., Song, F., Jiang, P., Li, Z. H., & Cai, W. Z. (2015). Higher‐level phylogeny of paraneopteran insects inferred from mitochondrial genome sequences. Scientific Reports, 5, 8527. 10.1038/srep08527 25704094 PMC4336943

[ece38001-bib-0022] Li, Z. Z., & Fan, Z. H. (2017). Coelidiinae (Hemiptera: Cicadellidae) from China (pp. 1–443). Guizhou Science and Technology Publishing House Press.

[ece38001-bib-0023] Liua, Y. Q., Song, F., Jiang, P., Wilsonc, J. J., Cai, W. Z., & Li, H. (2018). Compositional heterogeneity in true bug mitochondrial phylogenomics. Molecular Phylogenetics and Evolution, 118, 135–144. 10.1016/j.ympev.2017.09.025 28986237

[ece38001-bib-0024] Martin, A. P. (1995). Metabolic rate and directional nucleotide substitution in animal mitochondrial DNA. Molecular Biology and Evolution, 12, 1124–1131. 10.1006/jhev.1995.1071 8524045

[ece38001-bib-0025] Morgan, C. C., Foster, P. G., Webb, A. E., Pisani, D., McInerney, J. O., & O'Connell, M. J. (2013). Heterogeneous models place the root of the placental mammal phylogeny. Molecular Biology and Evolution, 30, 2145–2156. 10.1093/molbev/mst1 23813979 PMC3748356

[ece38001-bib-0026] Moritz, C., Dowling, T. E., & Brown, W. M. (1987). Evolution of animal mitochondrial DNA: Relevance for population biology and systematics. Annual Review of Ecology Evolution and Systematics, 18, 269–292. 10.1146/annurev.es.18.110187.001413

[ece38001-bib-0027] Nelson, L. A., Lambkin, C. L., Batterham, P., Wallman, J. F., Dowton, M., Whiting, M. F., Yeates, D. K., & Cameron, S. L. (2012). Beyond barcoding: A mitochondrial genomics approach to molecular phylogenetics and diagnostics of blowflies (Diptera: Calliphoridae). Gene, 511, 131–142. 10.1016/j.gene.2012.09.103 23043935

[ece38001-bib-0028] Nguyen, L. T., Schmidt, H. A., Arndt, V. H., & Minh, B. Q. (2014). IQ‐TREE: A fast and effective stochastic algorithm for estimating maximum‐likelihood phylogenies. Molecular Biology and Evolution, 32, 268–274. 10.1093/molbev/msu300 25371430 PMC4271533

[ece38001-bib-0029] Nielson, M. W. (2015). A revision of the tribe Coelidiini of the Oriental, Palearctic and Australian biogeographical regions (Hemiptera: Cicadellidae: Coelidiinae). Insecta Mundi, 0410, 1–202.

[ece38001-bib-0030] Perna, N. T., & Kocher, T. D. (1995). Patterns of nucleotide composition at fourfold degenerate sites of animal mitochondrial genomes. Journal of Molecular Evolution, 41, 353–358. 10.1007/BF00186547 7563121

[ece38001-bib-0031] Ronquist, F., Teslenko, M., van der Mark, P., Ayres, D. L., Darling, A., Höhna, S., Larget, B., Liu, L., Suchard, M. A., & Huelsenbeck, J. P. (2012). MrBayes 3.2: Efficient bayesian phylogenetic inference and model choice across a large model space. Systematic Biology, 61, 539–542. 10.1093/sysbio/sys029 22357727 PMC3329765

[ece38001-bib-0032] Schattner, P., Brooks, A. N., & Lowe, T. M. (2005). The tRNAscan‐SE, snoscan and snoGPS web servers for the detection of tRNAs and snoRNAs. Nucleic Acids Research, 33, 686–689. 10.1093/nar/gki366 PMC1160127 15980563

[ece38001-bib-0033] Sheffield, N. C., Song, H. J., Cameron, S. L., & Whiting, M. F. (2009). Nonstationary evolution and compositional heterogeneity in beetle mitochondrial phylogenomics. Systematic Biology, 58, 381–394. 10.1093/sysbio/syp037 20525592

[ece38001-bib-0034] Song, F., Li, H., Jiang, P., Zhou, X. G., Liu, J. P., Sun, C. H., Vogler, A. P., & Cai, W. Z. (2016). Capturing the phylogeny of holometabola with mitochondrial genome data and Bayesian site‐heterogeneous mixture models. Genome Biology and Evolution, 8, 1411–1426. 10.1093/gbe/evw086 27189999 PMC4898802

[ece38001-bib-0035] Song, N., Cai, W. Z., & Li, H. (2017). Deep‐level phylogeny of Cicadomorpha inferred from mitochondrial genomes sequenced by NGS. Scientific Reports, 7, 1–11. 10.1038/s41598-017-11132-0 28874826 PMC5585334

[ece38001-bib-0036] Song, N., Zhang, H., & Zhao, T. (2019). Insights into the phylogeny of Hemiptera from increased mitogenomic taxon sampling. Molecular Phylogenetics and Evolution, 137, 236–249. 10.1016/j.ympev.2019.05.009 31121308

[ece38001-bib-0037] Stothard, P., Grant, J. R., & Van, D. G. (2017). Visualizing and comparing circular genomes using the CGView family of tools. Briefings in Bioinformatics, 20, 1576–1582. 10.1093/bib/bbx081 PMC6781573 28968859

[ece38001-bib-0038] Sudhir, K., Glen, S., & Koichiro, T. (2016). Mega 7: Molecular evolutionary genetics analysis version 7.0 for bigger datasets. Molecular Biology and Evolution, 33, 1870–1874. 10.1093/molbev/msw054 27004904 PMC8210823

[ece38001-bib-0039] Tamura, K., Stecher, G., Peterson, D., Filipski, A., & Kumar, S. (2013). MEGA6: Molecular evolutionary genetics analysis version 6.0. Molecular Phylogenetics and Evolution, 30, 2725–2729. 10.1093/molbev/mst197 PMC3840312 24132122

[ece38001-bib-0040] Timmermans, M. J., Barton, C., Haran, J., Ahrens, D., Culverwell, C. L., Ollikainen, A., Vogler, A. P., Dodsworth, S., Foster, P. G., Bocak, L., & Vogler, A. P. (2015). Family‐level sampling of mitochondrial genomes in Coleoptera: compositional heterogeneity and phylogenetics. Genome Biology and Evolution, 8, 161–175. 10.1093/gbe/evv241 26645679 PMC4758238

[ece38001-bib-0041] Viraktamath, C. A., & Meshram, N. M. (2019). Leafhopper tribe Coelidiini (Hemiptera: Cicadellidae: Coelidiinae) of the Indian subcontinent. Zootaxa, 4653, 1–91. 10.11646/zootaxa.4653.1.1 31716852

[ece38001-bib-0042] Wang, J. J., Li, H., & Dai, R. H. (2017). Complete mitochondrial genome of *Taharana fasciana* (Insecta, Hemiptera: Cicadellidae) and comparison with other Cicadellidae insects. Genetica, 145, 593–602. 10.1007/s10709-017-9984-8 28913775

[ece38001-bib-0043] Wang, J. J., Wu, Y. F., Dai, R. H., & Yang, M. F. (2020). Comparative mitogenomes of six species in the subfamily Iassinae (Hemiptera: Cicadellidae) and phylogenetic analysis. International Journal of Biological Macromolecules, 149, 1294–1303. 10.1016/j.ijbiomac.2020.01.270 32004599

[ece38001-bib-0044] Wang, J.‐J., Yang, M.‐F., Dai, R.‐H., Li, H. U., & Wang, X.‐Y. (2018). Characterization and phylogenetic implications of the complete mitochondrial genome of Idiocerinae (Hemiptera: Cicadellidae). International Journal of Biological Macromolecules, 120, 2366–2372. 10.1016/j.ijbiomac.2018.08.191 30179694

[ece38001-bib-0045] Wang, X. Y., Fan, Z. H., Li, Z. Z., & Dai, R. H. (2019). Key to genera of Chinese Coelidiinae leafhoppers, with description a new species of the leafhopper genus *Baseprocessa* (Hemiptera: Auchenorrhyncha: Cicadellidae). Zootaxa, 4701, 454–460. 10.11646/zootaxa.4701.5.5 32229928

[ece38001-bib-0046] Wang, X. Y., Wang, J. J., & Dai, R. H. (2021). Mitogenomics of five *Olidiana* leafhoppers (Hemiptera: Cicadellidae: Coelidiinae) and their phylogenetic implications. PeerJ, 9, e11086. 10.7717/peerj.11086 33986976 PMC8086571

[ece38001-bib-0047] Wang, X. Y., Wang, J. J., Fan, Z. H., & Dai, R. H. (2019). Complete mitogenome of *Olidiana ritcheriina* (Hemiptera: Cicadellidae) and phylogeny of Cicadellidae. PeerJ, 7, e8072. 10.7717/peerj.8072 31788356 PMC6883956

[ece38001-bib-0048] Wolstenholme, D. R. (1992). Animal Mitochondrial DNA: Structure and Evolution. International Review of Cytology, 141, 173–216. 10.1016/S0074-7696(08)62066-5 1452431

[ece38001-bib-0052] Xue, Q. Q., Dietrich, C. H., & Zhang, Y. L. (2020). Phylogeny and classification of the leafhopper subfamily Eurymelinae (Hemiptera: Cicadellidae) inferred from molecules and morphology. Systematic Entomology, 45, 687–702. 10.1111/syen.12425

[ece38001-bib-0049] Yan, C., & Zu, Z. (2019). The complete mitochondrial genome of *Cosmoscarta dorsimacula* . Mitochondrial DNA Part B, 4(1), 975–976. 10.1080/23802359.2019.1580158 PMC7687497 33365449

[ece38001-bib-0050] Zhang, Y. L. (1990). A taxonomic study of Chinese Cicadellidae (Homoptera) (pp. 1–218). Tianze Publishing House Press.

