# Supplementary material for: Structural features of the mitogenome of the leafhopper genus Cladolidia (Hemiptera: Cicadellidae: Coelidiinae) and phylogenetic implications in Cicadellidae
Source: Ecol Evol. 2021 Aug 5;11(18):12554–66. doi: 10.1002/ece3.8001 (PMC8462178; doi:10.1002/ece3.8001)
Supplement: Supplementary file 1 — Appendix S1 [file ECE3-11-12554-s001.doc]

**TABLE S1** Specimen collection information.

| Name | Locality | Sample Number | Collection Time | Collector |
| --- | --- | --- | --- | --- |
| *Cladolidia biungulata* | Mt: Leigongshan, Guizhou province, China | GZUW-C-00082 | 08, August 2019 | Yalin Yao |
| *Cladolidia* *robusta* | Anzihe river nature reserve, Sichuan province, China | GZUW-C-00001 | 28, July 2018 | Hongpin Zhang |

**TABLE S2** Information of samples for phylogenetic analyses.

| **Taxa** | Genus | Genebank | Length | References |
| --- | --- | --- | --- | --- |
| **Cicadellinae** | *Bothrogonia tongmaiana* | NC049895 | 15,539 | Unpublished |
|  | *Cofana yasumatsui* | NC049087 | 15,019 | Unpublished |
|  | *Homalodisca coagulata* | AY875213 | 15,304 | Unpublished |
| **Coelidiinae** | *Cladolidia biungulata* | MW406474 | 15,247 | **This study** |
|  | *Cladolidia robusta* | MW406475 | 15,376 | **This study** |
|  | *Olidiana alata* | MN780581 | 15,205 | Wang, Wang & Dai, 2021 |
|  | *Olidiana longsticka* | MN780582 | 15,993 | Wang, Wang & Dai, 2021 |
|  | *Olidiana olbliquea* | MN780583 | 15,312 | Wang, Wang & Dai, 2021 |
|  | *Olidiana ritcheri* | MN780584 | 15,372 | Wang, Wang & Dai, 2021 |
|  | *Olidiana ritcheriina* | NC045207 | 15,166 | Wang et al., 2019 |
|  | *Olidiana* sp. | KY039119 | 15,253 | Song, Cai & Li, 2019 |
|  | *Olidiana tongmaiensis* | MN780585 | 15,363 | Wang, Wang & Dai, 2021 |
|  | *Taharana fasciana* | NC036015 | 15,161 | Wang et al., 2019a |
| **Deltocephalinae** | *Abrus expansivus* | NC045238 | 15,904 | Wang & Xing, 2019 |
|  | *Drabescus ineffectus* | NC050258 | 15,744 | Xu, Yu & Zhang, 2020 |
|  | *Macrosteles quadrimaculatus* | NC039560 | 15,734 | Du, Dietrich & Dai, 2019 |
|  | *Maiestas dorsalis* | NC036296 | 15,352 | Du et al., 2017 |
|  | *Paramacrosteles nigromaculatus* | NC045270 | 15,011 | Unpublished |
|  | *Roxasellana stellata* | NC050257 | 15,361 | Xu, Yu & Zhang, 2020 |
|  | *Scaphoideus varius* | KY817245 | 15,207 | Du, Dai & Dietrich, 2017 |
|  | *Yanocephalus yanonis* | NC036131 | 15,623 | Song, Cai & Li, 2017 |
| **Evacanthinae** | *Evacanthus acuminatus* | MK948205 | 14,793 | Wang, et al., 2019 |
|  | *Evacanthus interruptus* | MK251114 | 12,426 | Song, Cai & Li, 2019 |
|  | *Onukia onukii* | MK251119 | 12,395 | Song, Cai & Li, 2019 |
|  | *Sophonia linealis* | KX437723 | 13,296 | Song, Cai & Li, 2019 |
| **Iassinae** | *Batracomorphus lateprocessus* | NC045858 | 15,164 | Song, Cai & Li, 2019 |
|  | *Gessius rufidorsus* | MN577633 | 14,634 | Wang, et al., 2020 |
|  | *Iassus dorsalis* | MN577634 | 15,176 | Wang, et al., 2020 |
|  | *Krisna rufimarginata* | NC046068 | 14,724 | Wang, et al., 2020 |
|  | *Trocnadella arisana* | NC036480 | 15,131 | Wang, et al., 2020 |
| **Idiocerinae** | *Idioscopus myrica* | MH492317 | 15,393 | Dai, Wang & Yang, 2018 |
|  | *Idioscopus nitidulus* | NC029203 | 15,287 | Unpublished |
|  | *Idiocerus laurifoliae* | NC039741 | 16,811 | Wang et al., 2018 |
|  | *Liocratus salicis* | MG813490 | 16,436 | Wang et al., 2018 |
|  | [*Populicerus populi*](https://www.ncbi.nlm.nih.gov/nuccore/NC_039427.1) | NC039427 | 16,494 | Wang et al., 2018 |
|  | *Rhytidodus viridiflavus* | MN935488 | 16,842 | Di et al., 2020 |
| **Macropsinae** | *Macropsis notata* | NC042723 | 16,323 | Wang et al., 2020 |
|  | *Oncopsis nigrofasciata* | MG813492 | 15,927 | Wang et al., 2020 |
| **Megophthalminae** | *Durgades nigropicta* | NC035684 | 15,974 | Wang et al., 2017 |
|  | *Japanagallia spinosa* | NC035685 | 15,655 | Wang et al., 2017 |
| **Mileewinae** | *Mileewa margheritae* | MT483998 | 15,375 | He & Yang, 2020 |
|  | *Mileewa ponta* | MT497465 | 15,999 | He & Yang, 2020b |
|  | *Mileewa albovittata* |  |  |  |
|  | *Mileewa alara* |  |  |  |
| **Typhlocybinae** | *Empoasca flavescens* | MK211224 | 15,152 | Luo et al., 2019 |
|  | *Eupteryx minuscula* | MN910279 | 16,944 | Yang et al., 2020 |
|  | *Ghauriana sinensis* | MN699874 | 15,491 | Shi, Yu & Yang, 2020 |
|  | *Limassolla lingchuanensis* | NC046037 | 15,716 | Yuan et al., 2020 |
|  | *Mitjaevia protuberanta* | NC047465 | 15,472 | Yuan, Li & Song, 2020 |
|  | *Parazyginella tiani* | MT683891 | 17,562 | Unpublished |
|  | *Zyginella minuta* | MT488436 | 15,544 | Han et al., 2020 |
| **Ledrinae** | *Ledra auditura* | MK387845 | 14,918 | Wang et al., 2019 |
|  | *Petalocephala chlorophana* | KX437734 | 14,918 | Huan & Zhang, 2020 |
|  | *Tituria pyramidata* | NC046701 | 15,331 | Li & Dai, 2019 |
|  | *Tituria sagittata* | NC051528 | 14,918 | Huan & Zhang, 2020 |
| **Treehoppers** | *Entylia carinata* | NC033539 | 15,662 | Mao, Yang & Bennett, 2016 |
|  | *Hypsauchenia hardwickii* | NC044705 | 15,618 | Hu et al., 2019 |
|  | *Leptocentrus albolineatus* | NC044707 | 15,508 | Hu et al., 2019 |
|  | *Maurya qinlingensis* | NC044706 | 16,011 | Hu et al., 2019 |
|  | *Tricentrus brunneus* | NC044708 | 16,467 | Hu et al., 2019 |
|  | *Cosmoscarta bispecularis* | KP064511 | 15,426 | Han, Liu & Liang, 2014 |
|  | *Tettigades auropilosa* | KM000129 | 14,944 | Unpublished |

References

Du, Y.M., Zhang, C.N., Dietrich, C.H., & Zhang, Y.L., (2017). Dai, W. Characterization of the complete mitochondrial genomes of *Maiestas dorsalis* and *Japananus hyalinus* (Hemiptera: Cicadellidae) and comparison with other Membracoidea. *Sci. Rep.*, *7*(1): 14197.

Du, Y.M., Dietrich, C.H., & Dai, W. (2019). Complete mitochondrial genome of *Macrosteles quadrimaculatus* (Matsumura) (Hemiptera: Cicadellidae: Deltocephalinae) with a shared tRNA rearrangement and its phylogenetic implications. *Int. J. Biol. Macromol.*, *122*: 1027–1034.

Du, Y.M., Dai, W., & Dietrich, C.H. (2017). Mitochondrial genomic variation and phylogenetic relationships of three groups in the genus *Scaphoideus* (Hemiptera: Cicadellidae: Deltocephalinae). *Sci. Rep.*, *7*(1): 16908.

Han, C., Yan, B., Yu, X.F., & Yang, M.F. (2020). Complete mitochondrial genome of *Zyginella minuta* (cicadellidae: typhlocybinae: zyginellini) from china, with its phylogenetic analysis. *Mitochondrial DNA B*, *5*(3), 2795–2796.

Han, Y., Liu, J., & Liang, A.P. (2014). The complete mitochondrial genome of *Cosmoscarata bispecularis* (Hemiptera, Cicadomorpha, Cercopoidea, Cercopidae). *Dna Sequence*, *27*(6), 3957-3958.

He, H.L., & Yang, M.F. (2020a). The mitogenome of *Mileewa margheritae* (Hemiptera: Cicadellidae: Mileewinae). *Mitochondrial DNA B*, *5*(3), 3163-3164.

He, H.L., & Yang, M.F. (2020b). Characterization and phylogenetic analysis of the mitochondrial genome of *Mileewa ponta* (Hemiptera: Cicadellidae: Mileewinae). *Mitochondrial DNA B*, *5*(3), 2976–2977.

Hu, K., Yuan, F., Dietrich, C.H., & Yuan, X. Q. (2019). Structural features and phylogenetic implications of four new mitogenomes of centrotinae (hemiptera: membracidae). *Int. J. Biol. Macromol.*, *139*, 1018-1027.

Huan, W.H., & Zhang, Y.L. (2020). Characterization of two complete mitochondrial genomes of ledrinae (hemiptera: cicadellidae) and phylogenetic analysis. *Insects*, *11*(609).

Jiang, J., Yuan, X., Yuan, Z., & Song, Y. (2020). The complete mitochondrial genome of *Parathailocyba oral* (Hemiptera: Cicadellidae: Typhlocybinae). *Mitochondrial DNA B*, *5*(2), 1981–1982.

Li, D.F., & Dai, R.H. (2019). The complete mitochondrial genome of *Tituria pyramidata* (hemiptera: cicadellidae: ledrinae) from china. *Mitochondrial DNA B*, *5*(2), 1757–1758.

Li, H., Leavengood, J.M., Chapman, E.G., Burkhardt, D., Song, F., & Jiang, P. (2017). Mitochondrial phylogenomics of hemiptera reveals adaptive innovations driving the diversification of true bugs. *P. Roy. Soc B-Biol. Sci.*, *284*(1862), 20171223.

Liu, J.H., Sun, C.Y., Long, J., & Guo, J.J. (2017). Complete mitogenome of tea green leafhopper, *Empoasca onukii* (Hemiptera: Cicadellidae) from Anshun, Guizhou Province in China. *Mitochondrial DNA B*, *2*(2): 808–809.

Mao, M., Yang, X., & Bennett, G. (2016). The complete mitochondrial genome of *Entylia carinata* (hemiptera: membracidae). *Mitochondrial DNA B*, *1*(1), 662-663.

Song, N., Cai, W.Z., & Li, H. (2017). Deep-level phylogeny of Cicadomorpha inferred from mitochondrial genomes sequenced by NGS. *Sci. Rep.*, *7*, 1–11.

Song, N., Cai, W.Z., & Li, H. (2019). Insights into the phylogeny of Hemiptera from increased mitogenomic taxon sampling. *Mol. Phylogenet. Evol.*, *137*,236–249.

Wang, J.J., Dai, R.H., Li, H., & Zhan, H.P. (2017). Characterization of the complete mitochondrial genome of *Japanagallia spinosa* and *Durgades nigropicta* (Hemiptera: Cicadellidae: Megophthalminae). *Biochem. Syst. Ecol*., *74*: 33–41.

Wang, J.J., Li, D.F., Li, H., Yang, M.F., & Dai, R.H. (2019). Structural and phylogenetic implications of the complete mitochondrial genome of *Ledra auditura*. *Sci. Rep.*, 1, 15746.

Wang, J.J., Li, H., & Dai, R.H. (2017). Complete mitochondrial genome of *Taharana fasciana* (Insecta, Hemiptera: Cicadellidae) and comparison with other Cicadellidae insects. *Genetica*, *145*, 593–602.

Wang, J.J., Wu, Y. F., Dai, R.H., & Yang, M.F. (2020). Comparative mitogenomes of six species in the subfamily Iassinae (Hemiptera: Cicadellidae) and phylogenetic analysis. *Int. J. Biol. Macromol.*, *149*, 1294–1303.

Wang, J.J., Wu, Y.F., Yang, M.F., & Dai, R.H. (2020). The phylogenetic implications of the mitochondrial genomes of *macropsis notata* and *oncopsis nigrofasciata*. *Frontiers in Genetics*, *11*, 443.

Wang, J.J., & Xing, J.C. (2019). Complete mitochondrial genome of *Abrus expansivus* (Hemiptera: Cicadellidae: Deltocephalinae) from China. *Mitochondrial DNA B*, *4*(1): 197–198.

Wang, J.J., Yang, M.F., Dai, R.H., & Wang, X.Y. (2018). Characterization and phylogenetic implications of the complete mitochondrial genome of Idiocerinae (Hemiptera: Cicadellidae). *Int. J. Biol. Macromol.*, *120*, 2366–2372.

Wang, X.Y., Wang, J.J., Fan, Z.H., & Dai, R.H. (2019). Complete mitogenome of *Olidiana ritcheriina* (Hemiptera: Cicadellidae) and phylogeny of Cicadellidae. *PeerJ*, *7*, e8072.

Wang X.Y., Wang J.J., & Dai R.H. (2021). Mitogenomics of five *Olidiana* leafhoppers (Hemiptera: Cicadellidae: Coelidiinae) and their phylogenetic implications. *PeerJ*, *9*:e11086.

Xu, D., Yu, T., & Zhang, Y.L. (2020). Characterization of the Complete Mitochondrial Genome of *Drabescus ineffectus* and *Roxasellana stellata* (Hemiptera: Cicadellidae: Deltocephalinae: Drabescini) and Their Phylogenetic Implications. *Insects*, *11*(8), E534.

Yuan, Z., Yang, X., Li, C., & Song, Y. (2019). The complete mitochondrial genome of the leafhopper *Evacanthus acuminatus* (Hemiptera: Cicadellidae: Evacanthinae). *Mitochondrial DNA B*, *4*(2), 3866–3867.

Zhou, X., Dietrich, C.H., & Huang, M. (2020). Characterization of the complete mitochondrial genomes of two species with preliminary investigation on phylogenetic status of Zyginellini (Hemiptera: Cicadellidae: Typhlocybinae). *Insects*, *11*(10), 684.

**TABLE S3** The best partitioning scheme selected by Partition Finder for two (13PCGs12 and AA) datasets.

| Datasets | Subset | Best Model | Site (bp) | Partition names |
| --- | --- | --- | --- | --- |
| 13PCGs12 | 1 | GTR+I+G | 743 | cox3_pos1, cox2_pos1, atp8_pos2, atp6_pos1 |
|  | 2 | TVM+I+G | 1055 | nad6_pos2, nad3_pos2, nad2_pos2, cox3_pos2, atp6_pos2 |
|  | 3 | GTR+I+G | 1586 | nad2_pos1, nad6_pos1, nad3_pos1, atp8_pos1, nad5_pos1, nad4_pos1 |
|  | 4 | GTR+I+G | 869 | cox1_pos1, cob_pos2 |
|  | 5 | TVM+I+G | 735 | cox2_pos2, cox1_pos2 |
|  | 6 | GTR+I+G | 669 | cob_pos1, nad1_pos1 |
|  | 7 | GTR+I+G | 1263 | nad4_pos2, nad5_pos2, nad1_pos2 |
|  | 8 | GTR+G | 164 | nad4l_pos2, nad4l_pos1 |
| AA | 1 | MTART+I+G+F | 1328 | atp8, nad6, nad3, cox3, atp6, nad2, cox2 |
|  | 2 | MTART+I+G+F | 869 | cob, cox1 |
|  | 3 | MTART+I+G+F | 1345 | nad4, nad5, nad1, nad4l |


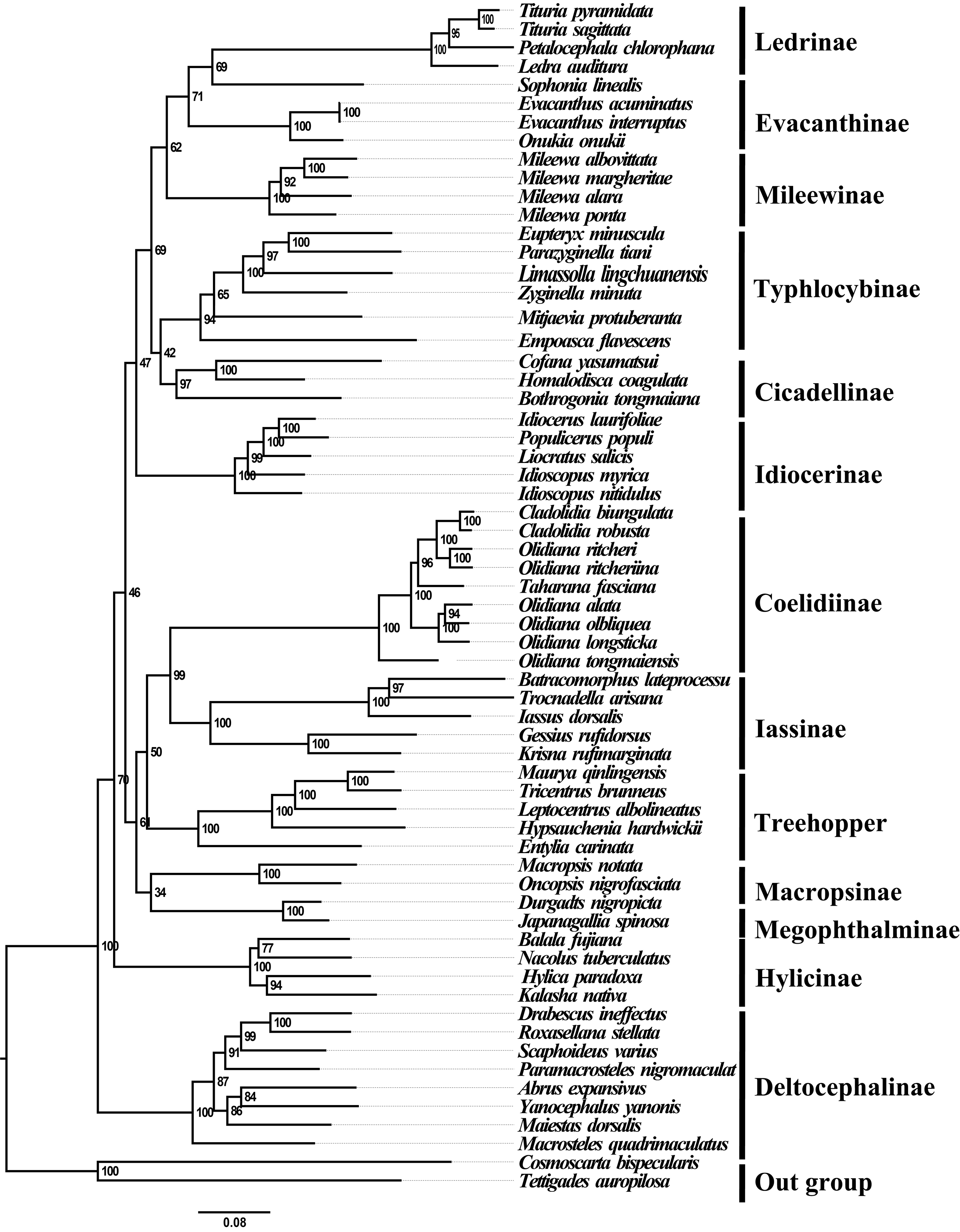


FIGURE S1. Phylogenetic tree Cicadellidae species inferred via maximum likelihood analyses of the PCG12 datasets.


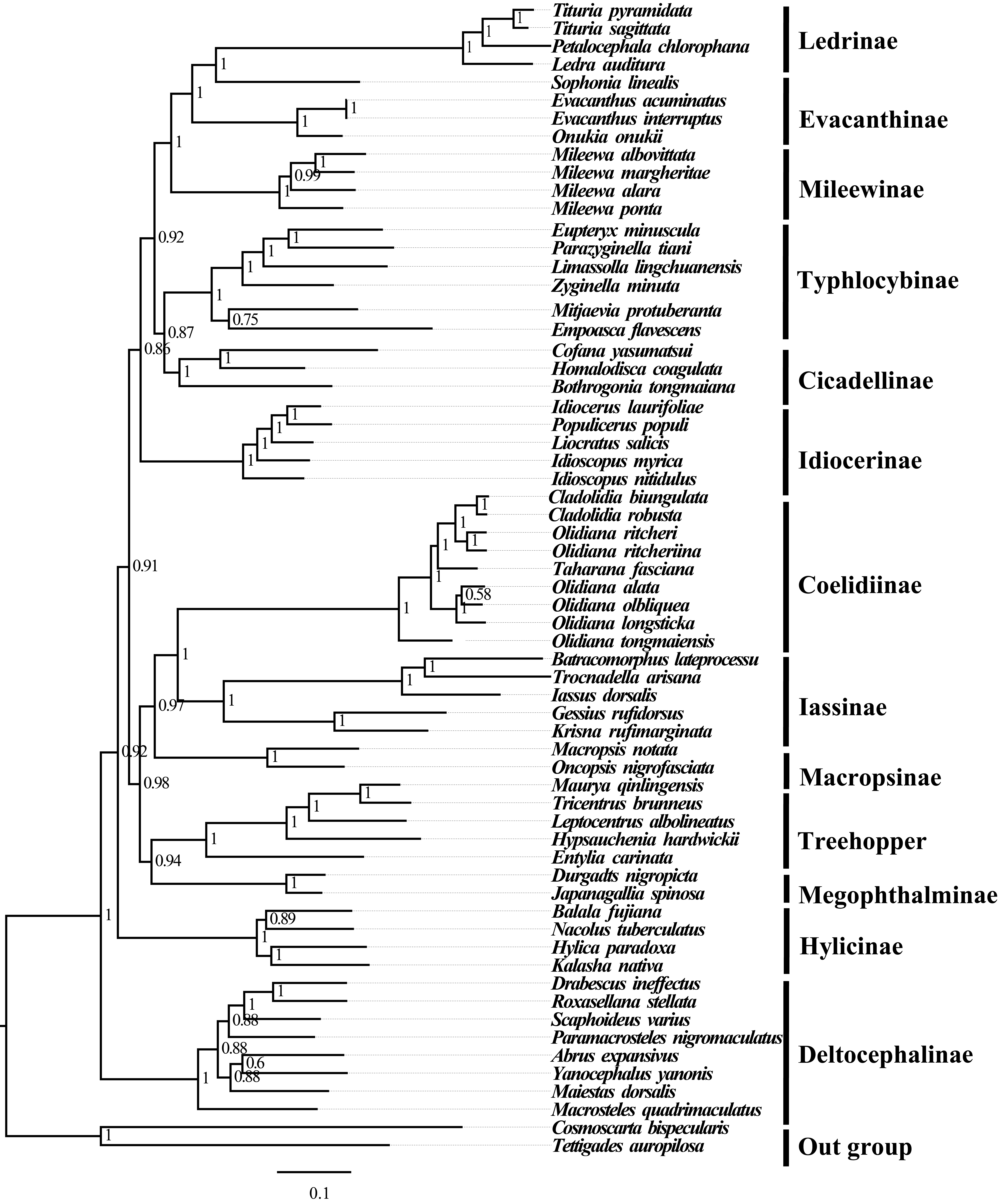


FIGURE S2. Phylogenetic tree of Cicadellidae species inferred via Bayesian analyses of the PCG12 datasets.
